# CD8^+^ T-Cell Responses in Acute Hepatitis C Virus Infection

**DOI:** 10.3389/fimmu.2014.00266

**Published:** 2014-06-06

**Authors:** Pil Soo Sung, Vito Racanelli, Eui-Cheol Shin

**Affiliations:** ^1^Laboratory of Immunology and Infectious Diseases, Graduate School of Medical Science and Engineering, Korea Advanced Institute of Science and Technology, Daejeon, South Korea; ^2^Department of Internal Medicine and Clinical Oncology, University of Bari Medical School, Bari, Italy

**Keywords:** hepatitis C virus, acute infection, outcome, CD8^+^ T-cell, function

## Abstract

Hepatitis C virus (HCV) infects approximately 170 million people worldwide and is a major cause of life-threatening liver diseases such as liver cirrhosis and hepatocellular carcinoma. Acute HCV infection often progresses to chronic persistent infection, although some patients recover spontaneously. The divergent outcomes of acute HCV infection are known to be determined by differences in virus-specific T-cell responses among patients. Of the two major T-cell subsets, CD8^+^ T-cells are known to be the key effector cells that control viral infections via cytolytic activity and cytokine secretion. Herein, we review various aspects of HCV-specific CD8^+^ T-cell responses in acute HCV infection. In particular, we focus on timing of CD8^+^ T-cell responses, relationship between CD8^+^ T-cell responses and outcomes of acute HCV infection, receptor expression on CD8^+^ T-cells, breadth of CD8^+^ T-cell responses, and viral mutations.

## Introduction

Hepatitis C virus (HCV) is a positive-stranded RNA virus of the genus *Hepacivirus* in the family *Flaviviridae* that infects approximately 170 million people worldwide (1, 2). Acute HCV infection is spontaneously cleared in 20–30% of patients; however, the majority of patients fail to clear HCV and develop chronic persistent infection, which tends to progress to life-threatening liver diseases such as liver cirrhosis and hepatocellular carcinoma (3). Typically, patients with self-limited acute HCV infection undergo sustained viral clearance within the first 12 weeks of disease onset, whereas viremia beyond 6 months generally indicates chronic evolution (4). The divergent outcomes of acute HCV infection are known to be determined by differences in virus-specific T-cell responses among patients (5–9).

Of the two major T-cell subsets, CD8^+^ T-cells are known to be the key effector cells that control viral infections via cytolytic activity and cytokine secretion. The importance of cytolytic function in HCV infection is suggested by the fact that CD8^+^ T-cell responses coincide not only with the decrease of HCV RNA titers in blood, but also with the peak in serum alanine aminotransferase (ALT) (10, 11), which is a marker of hepatocyte injury. Interferon-γ (IFN-γ) secreted by CD8^+^ T-cells also directly exerts antiviral functions. It was shown that IFN-γ suppresses viral replication in HCV replicon studies (12, 13). In addition, the antiviral functions of CD8^+^ T-cell-secreted IFN-γ were further demonstrated in cocultures of HCV replicon cells and HCV-specific CD8^+^ T-cells (14).

In fact, the initial study on immune response in HCV-infected chimpanzees showed that HCV-specific CD8^+^ T-cell response well correlated with protection against HCV (15). The role of CD8^+^ T-cells in HCV infection was once clearly demonstrated by a chimpanzee study with antibody-mediated depletion of CD8^+^ T-cells. In this study, CD8^+^ T-cells were depleted prior to HCV challenge, and viral load remained at high levels for a prolonged period without CD8^+^ T-cells (16). Viral clearance was only achieved after HCV-specific CD8^+^ T-cells recovered in the liver (16).

The importance of CD8^+^ T-cells in HCV infection is also supported by immunogenetics studies that showed associations between specific human leukocyte antigen (HLA) class I allotypes and clinical outcome of HCV infection. HLA-B27, -B57, and -A3 are known to be associated with a high rate of HCV clearance (17–19).

In this review, we summarize various aspects of CD8^+^ T-cell responses in acute HCV infection. In particular, we focus on timing of CD8^+^ T-cell responses, relationship between CD8^+^ T-cell responses and outcomes of acute HCV infection, receptor expression on CD8^+^ T-cells, breadth of CD8^+^ T-cell responses, and viral mutations.

## Clinical Course of Acute HCV Infection

In principle, acute HCV infection is defined as the first 6 months following infection with HCV. Although HCV RNA titer is high in the circulation, the majority of patients remain asymptomatic during acute HCV infection with only 15% of infected patients undergoing symptomatic acute hepatitis C (20). In addition, a third of symptomatic infected patients do not produce detectable anti-HCV antibodies at the onset of symptoms (21). Accordingly, it is difficult to identify patients with acute HCV infection and to study immune responses during this period. Therefore, much of the knowledge regarding T-cell responses in acute HCV infection has been gleaned through studies of a chimpanzee model. Since the initial study on CD8^+^ T-cells in chimpanzees with acute HCV infection (15), monoclonal HCV infection of chimpanzees further enabled precise analyses of CD8^+^ T-cells during acute HCV infection (11, 22).

After inoculation of 100 chimpanzee infectious dose 50 (CID50) of monoclonal HCV-positive plasma, viral RNA becomes detectable in serum within 1–2 weeks and increases rapidly thereafter (11). However, liver injury only increases minimally despite a high level of viremia during this incubation phase, which lasts about 8–12 weeks. During the incubation period, the expression of IFN-stimulated genes (ISGs) is induced in the infected liver (23). In terms of ISGs induction, HCV infection contrasts with hepatitis B virus (HBV) infection. In fact, ISGs expression is not induced by HBV infection (24), and thus HBV is considered a stealth virus (25).

**Figure 1:**
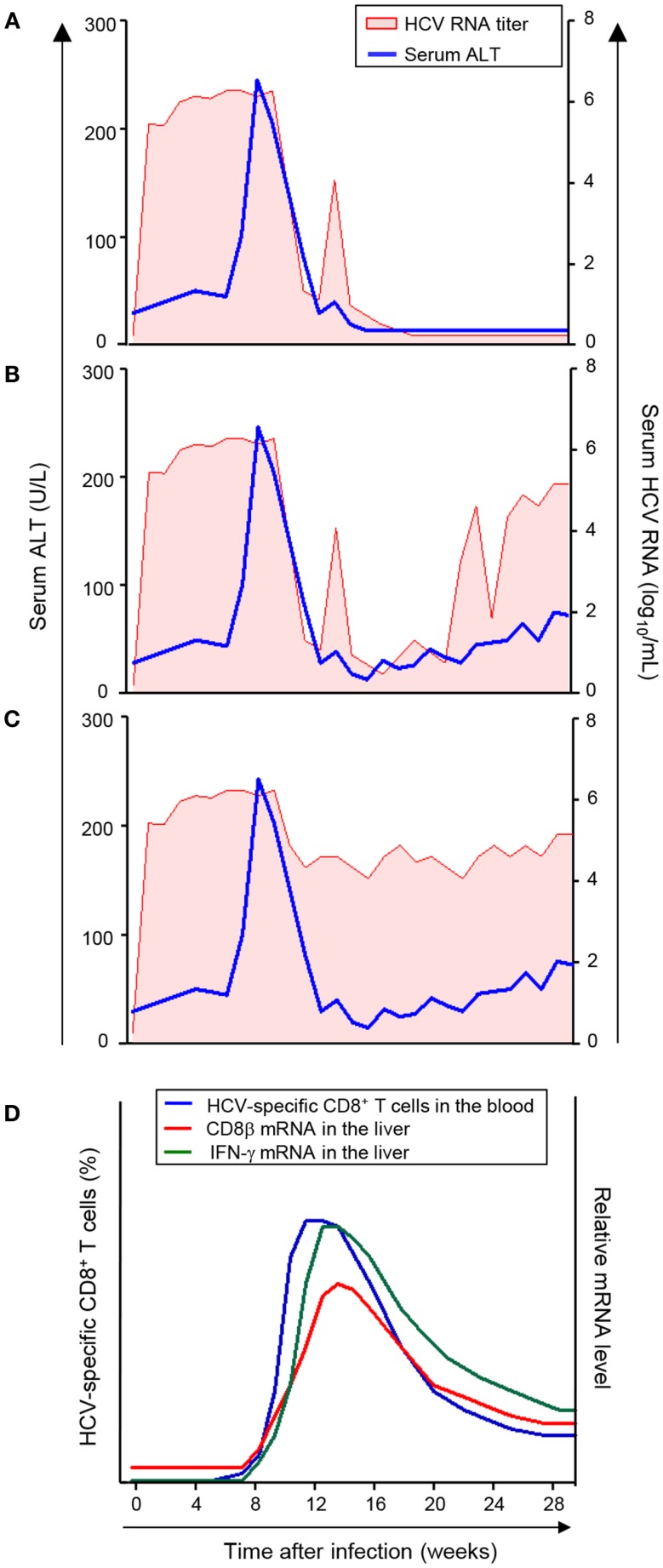
**Virologic courses and kinetics of CD8^+^ T-cell responses in acute HCV infection**. **(A–C)** Three virologic patterns of acute HCV infection (11). **(D)** Kinetics of CD8^+^ T-cell response in acute HCV infection (20, 21). The frequency of HCV-specific CD8^+^ T-cells in the blood and mRNA levels of CD8β and IFN-γ in the liver are presented. The appearance of HCV-specific CD8^+^ T-cells in the blood coincides with the increase in mRNA levels of CD8β and IFN-γ in the liver and with serum ALT elevation and a decrease in HCV RNA titer.

Serum ALT peaks at 8–12 weeks after HCV infection, and the viral RNA titer begins to decrease at this time (11). Of interest, there are three different patterns of viral RNA decrease as initially proposed by Thimme et al. (10). In the first pattern, HCV RNA titer is decreased below the lower detection limit, and ultimately HCV is cleared (Figure 1A). In the second pattern, HCV RNA titer is decreased below the lower detection limit, but HCV RNA reappears in the serum within several weeks and the infection evolves to a chronic persistent infection (Figure 1B). In the third pattern, HCV RNA titer only undergoes a 2–3 log reduction and the infection evolves to a chronic persistent infection (Figure 1C). Thus, HCV infection of chimpanzees with a single molecular clone results in a relatively uniform course of acute HCV infection with significantly divergent outcomes. Therefore, several interesting immunological questions have arisen and have been studied in this model as described below.

## Timing of CD8^+^ T-Cell Responses in the Liver and Blood

Initially, a relatively late appearance of virus-specific T-cells in the infected liver was described during acute HCV infection in a chimpanzee study (10). Following infection, HCV replicates rapidly and viral titers increase to high levels in the circulation within days in a chimpanzee model (11, 26). However, HCV-specific T-cell responses do not become detectable in the liver until 8–12 weeks after infection (10). This delay of intrahepatic T-cell responses was shown through functional assays using *in vitro* expanded, liver-infiltrating T-cells (10). In addition, mRNA analyses showed that the expression of CD8β and IFN-γ increases in the liver at 8–12 weeks after infection (Figure 1D) (10, 11, 26, 27). Further, CD8β and IFN-γ mRNA expression in the liver coincides with a decline in viral titer (Figure 1D), even in the hosts with a chronically evolving course of infection (28).

Recently, the underlying cause of the delayed intrahepatic T-cell responses was revealed through study of HCV-infected chimpanzees (29). In this study, appearance of HCV-specific CD8^+^ T-cells in the circulation was analyzed using major histocompatibility complex (MHC) class I tetramers. During acute HCV infection, virus-specific CD8^+^ T-cells appear in the blood at late time points (8–12 weeks after HCV infection) and the appearance of HCV-specific CD8^+^ T-cells in the blood coincided precisely with the CD8^+^ T-cell infiltration to the liver, which was quantified by assessing CD8β mRNA levels (Figure 1D) (29). Furthermore, while HCV-specific CD8^+^ T-cells are induced at late time points, T-cell-recruiting chemokines for CXCR3 and CCR5 are expressed in the HCV-infected liver at early time points (2–8 weeks after HCV infection) (29). Thus, intrahepatic chemokines recruit HCV-specific CD8^+^ T-cells to the liver as soon as they appear in the blood. Therefore, it can be concluded that the delay in intrahepatic T-cell responses is not due to delayed recruitment, but rather, to delayed priming of HCV-specific CD8^+^ T-cells (29).

Thus, compared with other viral infections, HCV infection is characterized by delayed induction of virus-specific CD8^+^ T-cells. This delayed T-cell induction explains the long incubation period prior to development of immune-mediated liver injury (29). Moreover, it may explain why the rate of chronic evolution is so high after HCV infection (10, 11); however, the underlying cause of this delayed induction of HCV-specific CD8^+^ T-cells is not yet understood. During viral infection, efficient priming of CD8^+^ T-cells depends on cell death of virus-infected cells and the generation of inflammatory milieu. Virus-specific CD8^+^ T-cells are primed by cross presentation of viral antigens from virus-infected dying cells (30). HCV is known to be non-cytopathic, therefore it may not kill enough hepatocytes to release viral antigens and may not cause sufficient inflammation to induce cross presentation to CD8^+^ naïve precursor T-cells. This possibility needs to be investigated in further studies.

**Figure 2:**
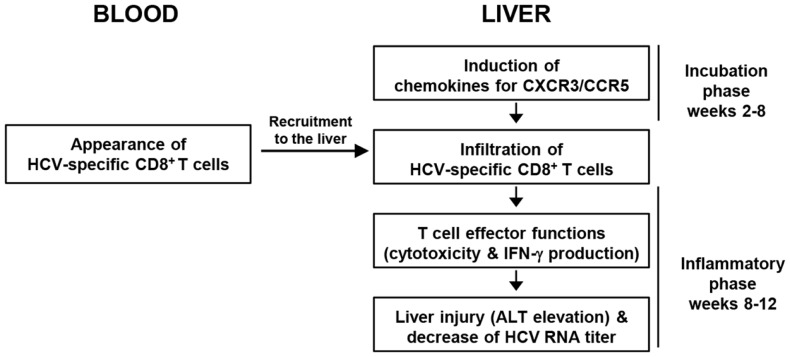
**Sequence of events during HCV-specific CD8^+^ T-cell responses in blood and liver throughout acute HCV infection**.

Figure 2 summarizes the sequence of major events in acute HCV infection. T-cell-recruiting chemokines for CXCR3 and CCR5 are produced in the liver 2–8 weeks after HCV infection. However, HCV-specific CD8^+^ T-cells appear in the blood no earlier than 8–12 weeks after infection. These cells express CXCR3 and/or CCR5 (29) and are immediately recruited to the liver. Intrahepatic infiltration of T-cells results in liver injury, manifested by serum ALT elevation and a decrease in HCV RNA titer.

The course of acute HCV infection is modified after re-infection of previously recovered chimpanzees. The duration of the re-infection is substantially shorter than that of the primary infection even after re-challenge with different HCV genotypes (31, 32). In addition, HCV-specific T-cell responses in the blood and liver are detected at much earlier time point after re-infection, compared to the primary infection (33), indicating rapid recall responses of HCV-specific memory T-cells. In a setting of re-infection, the role of CD8^+^ T-cells was studied by antibody-mediated depletion of CD8^+^ T-cells. Depletion of CD8^+^ T-cells and subsequent HCV re-challenge resulted in prolonged viremia, and viral clearance occurred when HCV-specific CD8^+^ T-cells recovered in the liver (16).

**Figure 3:**
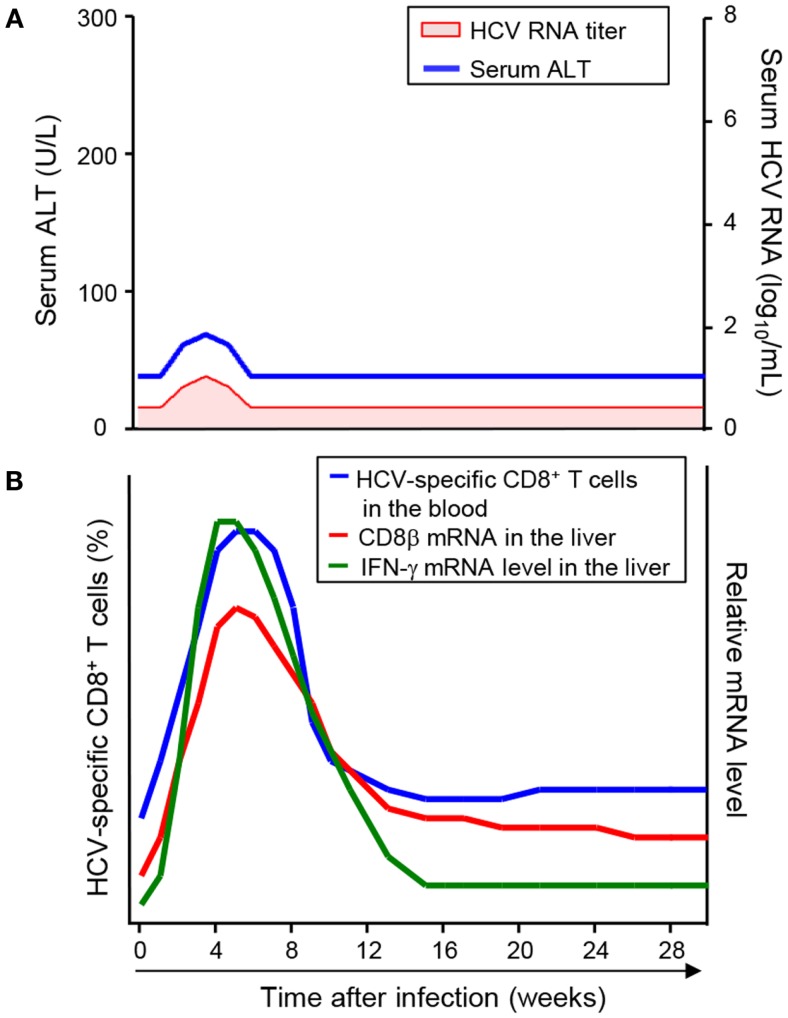
**Virologic course and kinetics of CD8^+^ T-cell responses after HCV challenge in successfully vaccinated hosts**. **(A)** Virologic course after HCV challenge in successfully vaccinated hosts (23). HCV RNA was transiently detected and disappeared, and serum ATL was also transiently elevated. **(B)** Kinetics of CD8^+^ T-cell responses after HCV challenge in successfully vaccinated hosts (19, 24). Early CD8^+^ T-cell responses are shown by the frequency of HCV-specific CD8^+^ T-cells in the blood and mRNA levels of CD8β and IFN-γ in the liver.

In line with the re-infection studies, successful vaccination shifts the timing of HCV-specific CD8^+^ T-cell responses earlier. In a study of chimpanzees vaccinated with recombinant adenovirus and DNA against HCV non-structural proteins, HCV RNA was transiently detected and disappeared, and serum ALT was also transiently increased (Figure 3A) (34). In these vaccinated hosts, HCV-specific CD8^+^ T-cells were detectable in the circulation after vaccination, and they were further expanded after HCV challenge (35). As a result, the frequency of HCV-specific CD8^+^ T-cells in the vaccinated animals reached peak levels as early as 4 weeks after HCV challenge (Figure 3B), which was 7–15 weeks earlier than in the mock-vaccinated control animals. Accordingly, intrahepatic T-cell responses, evaluated by mRNA levels of CD8β and IFN-γ, were much earlier in the vaccinated animals than in the mock-vaccinated control animals (Figure 3B) (27).

## HCV-Specific CD8^+^ T-Cell Responses and Acute HCV Infection Outcomes

As described above, HCV-specific CD8^+^ T-cells are induced and appear in the blood at 8–12 weeks after HCV infection and are recruited to the liver at the same time. However, the delayed induction of HCV-specific CD8^+^ T-cells was observed irrespective of the outcome of acute HCV infection (29). Instead, spontaneous resolution of acute HCV infection correlates with robust and sustained responses of HCV-specific T-cells in patients (6, 8, 36) and chimpanzees (10, 15). In contrast, chronic evolution of acute HCV infection is associated with weak and transient responses of HCV-specific T-cells (8, 10, 15, 36). In addition, the appearance of vigorous HCV-specific CD8^+^ T-cell responses against multiple epitopes was associated with the onset of viral clearance (6, 8). In a recent study of a chimpanzee model infected with monoclonal HCV, a similar result was reported (37). In this study, HCV-specific T-cell responses in the blood were comprehensively evaluated by IFN-γ enzyme-linked immunosorbent spot (ELISpot) assays with overlapping peptides covering all HCV proteins. While the HCV-specific IFN-γ response was strong in the chimpanzees with a self-limiting course of infection, it was undetectable or delayed in the chimpanzees with a chronically evolving course of infection (37). T-cell response was analyzed also in human immunodeficiency virus-positive patients with acute HCV infection, and spontaneous clearance of acute HCV infection was associated with strong T-cell response against HCV antigens (38).

Early antiviral therapy with pegylated IFN-α and ribavirin is effective in patients with acute HCV infection, and thus it prevents the development of chronic hepatitis. Then what will happen to HCV-specific CD8^+^ T-cells during successful antiviral therapy in patients with acute HCV infection? HCV-specific CD8^+^ T-cells observed in the early acute phase of HCV infection are maintained by continuous stimulation with HCV antigens; therefore, initiation of early antiviral therapy during acute HCV infection results in a rapid decline in CD8^+^ T-cell responses (39, 40). This finding implies that most HCV-specific CD8^+^ T-cells in the early acute phase of HCV infection are short-lived and furthermore, they are antigen-dependent effector cells rather than self-renewing memory T-cells (40).

As described above, acute HCV infection is often asymptomatic and induces mild inflammation in the liver. In rare cases, however, acute HCV infection results in severe hepatitis, which is known to be mediated by immune responses. In an interesting report regarding two rare severe cases of acute HCV infection, vigorous CD8^+^ T-cell responses were found to be narrowly focused against HCV NS3_1073_ peptide. Further, HCV NS3_1073_-specific CD8^+^ T-cells were cross-reactive with a peptide from influenza virus (41), though it was shown later that this cross-reactivity is relatively weak (42). It was inferred that influenza virus peptide-specific CD8^+^ memory T-cells generated during the previous influenza infection were vigorously activated due to cross-reactivity with HCV and subsequently induced severe liver damage (43). One striking feature of the clinical courses of these two patients was the development chronic HCV infection after severe acute infection in spite of the fact that they exhibited vigorous T-cell responses (41). This finding suggests that the vigor of HCV-specific CD8^+^ T-cells is not the sole factor determining the outcome of acute HCV infection.

## Receptor Expression on HCV-Specific CD8^+^ T-Cells

In chronic HCV infection, HCV-specific T-cells are functionally impaired, as evidenced by decreased proliferation, cytokine production, and cytolytic activity (44, 45). This impaired function has been attributed to upregulation of inhibitory receptors such as programed cell death-1 (PD-1) (46–53), cytotoxic T-lymphocyte-associated antigen 4 (CTLA-4) (47, 48), T-cell immunoglobulin and mucin domain-containing molecule 3 (Tim-3) (49, 50), 2B4, CD160, and killer cell lectin-like receptor G1 (KLRG1) (51). In chronically HCV-infected patients, HCV-specific CD127^lo^CD8^+^ T-cells co-expressed the inhibitory receptors PD-1, 2B4, CD160, and KLRG1 (51), and their expression was associated with the absence of viral escape mutations within the corresponding epitopes, indicating that continuous antigenic stimulation is required for the expression of the inhibitory receptors (51). In this report, HCV-specific CD127^lo^CD8^+^ T-cells could be partly, but not completely, restored functionally by PD-1 blockade alone, suggesting the role of multiple inhibitory receptors in T-cell dysfunction during chronic HCV infection (51).

However, the expression and role of inhibitory receptors has been controversial in acute HCV infection. Initially, high levels of PD-1 on HCV-specific T-cells and PD-1 mRNA in the liver were reported in patients (54) and chimpanzees (55) with acute HCV infection that later progressed to chronic persistent infection. In contrast, other studies showed that PD-1 expression is high on HCV-specific T-cells in acute HCV infection irrespective of the outcome of infection (56, 57). A recent study focused on this issue using chimpanzees acutely infected with monoclonal HCV (37). In this study, the PD-1 level on HCV-specific CD8^+^ T-cells peaked after appearance of HCV-specific CD8^+^ T-cells in the blood and decreased thereafter irrespective of the outcome of infection. In addition, intrahepatic mRNA levels of inhibitory receptor genes did not differ among chimpanzees with divergent outcomes of infection. Moreover, upregulation of PD-1 on HCV-specific CD8^+^ T-cells during acute HCV infection did not interfere with HCV clearance in spontaneously recovered hosts. Thus, following functional analyses of HCV-specific CD8^+^ T-cells, it was proposed that PD-1 is an activation marker, rather than an exhaustion marker, in acute HCV infection. This is consistent with the fact that PD-1 expression can be transiently induced by T-cell receptor and/or cytokine stimulation (58, 59).

In contrast to naïve chimpanzees, vaccinated chimpanzees have a different expression profile of PD-1 during acute HCV infection. In a study of chimpanzees vaccinated with recombinant adenovirus and DNA against HCV non-structural proteins, HCV-specific CD8^+^ T-cells in vaccinated animals displayed lower levels of PD-1 than those in the mock-vaccinated control animals (35). It was interpreted that early control of viremia by vaccine-induced T-cells prevented upregulation of PD-1 on HCV-specific CD8^+^ T-cells. Hence, the low expression of PD-1 might be an indicator of early viral control in vaccinated hosts (35). Taken together, it seems that the functional significance of PD-1 expression on virus-specific CD8^+^ T-cells differs between acute and chronic HCV infections, and between HCV infections with or without vaccination.

Interestingly, the outcome of acute HCV infection was related to the expression of CD127, also known as IL-7Rα, a marker of memory precursor cells (60). Indeed, a high frequency of CD127^+^ cells among HCV-specific CD8^+^ T-cells was observed in hosts that subsequently cleared the acute infection even when virus had been detectable in the blood. However, the frequency of CD127^+^ cells was low in hosts with chronically evolving infection. This is consistent with the fact that CD127 expression is decreased on HCV-specific T-cells in patients who were studied late in the course of acute HCV infection (9, 51, 61–63). Taken together, the frequency of HCV-specific CD127^+^CD8^+^ T-cells predicts the outcome of acute HCV infection when hosts are still viremic (37).

## Breadth of CD8^+^ T-Cell Responses and Viral Mutation

The breadth of CD8^+^ T-cell responses is an important factor in viral clearance during acute HCV infection (6, 64). Multiple epitopes-specific CD8^+^ T-cell responses contribute to viral clearance by suppressing the emergence of viral mutations. In fact, HCV-specific CD8^+^ T-cell responses are narrow over the chronically evolving course of acute HCV infection (6, 65). In contrast, a prospective study with chimpanzees showed that the animals with self-limited acute HCV infection exhibited CD8^+^ T-cell responses against multiple epitopes (10). During acute HCV infection, the broadest CD8^+^ T-cell responses occur during the early phase with a subsequent decline in the breadth of response (64, 66). For example, in a longitudinal study of acutely infected injection drug users, patients with progression to chronic infection lost recognition of one or more T-cell epitopes recognized during acute infection (64).

Acute HCV infection progresses to a chronic persistent infection if rapidly generated viral mutations overwhelm and outpace the CD8^+^ T-cell response. In the initial study with HCV-infected chimpanzees, it was demonstrated that the emergence of viral escape mutants in CD8^+^ T-cell epitopes correlated with viral persistence (67). A more recent study defined escape mutations in multiple CD8^+^ T-cell epitopes in patients that developed chronic infection. In this study, patients that cleared HCV had no substitutions within any recognized T-cell epitopes after the initial viremia (68).

During acute HCV infection, virus evolution is driven primarily by positive selection pressure exerted by CD8^+^ T-cells. This influence of immune pressure on viral evolution appears to subside as chronic infection is established (69). Recently, it was reported that the immunoregulatory changes of pregnancy reduce the selective pressure of HCV-specific CD8^+^ T-cells on T-cell epitopes, thereby facilitating vertical transmission of viruses with optimized replicative fitness (70). Thus, a strong CD8^+^ T-cell response eradicates HCV infection and a weak response will cause few mutations with optimized replication fitness. An intermediate immune response, which is usually the case, can stress the virus and select for escape mutants, resulting in viral persistence and a broader viral quasispecies (71).

## Conclusion

In this review, we have summarized various aspects of CD8^+^ T-cell responses in acute HCV infection. Acute HCV infection is characterized by delayed induction of virus-specific CD8^+^ T-cells, which cause the late onset of inflammation and viral control. In acute HCV infection, the expression of inhibitory receptors such as PD-1 is not related to T-cell exhaustion, while the expression of CD127 predicts the outcome of infection. In addition, the strength and breadth of HCV-specific CD8^+^ T-cell responses are associated with the outcome of acute HCV infection.

In previous studies, the nature of HCV-specific CD8^+^ T-cells has been examined using T-cells obtained from patients or chimpanzees with HCV infection. However, HCV-infected cells also need to be studied in the context of interaction between effector CD8^+^ T-cells and target cells. In particular, the regulation of the antigen processing and presentation machineries in HCV-infected cells is of interest, including immunoproteasomes (28), proteasome activators (72), and MHC molecules. In fact, a recent study demonstrated that HCV attenuates IFN-induced expression of MHC class I molecules, which are required for the recognition of virus-infected cells by CD8^+^ T-cells. This was suggested as a mechanism of evasion from antiviral CD8^+^ T-cell responses (73).

Currently, T-cell vaccines against HCV infection are now being developed. In particular, a result of phase I study on recombinant adenoviral vector-based vaccines was recently reported (74). Adenoviral vectors expressing non-structural proteins induced T-cell responses against multiple HCV proteins, and vaccine-induced T-cells recognized heterologous strains. Moreover, they were polyfunctional and sustained for a prolonged period (74). These vaccine candidates might be useful for both prophylactic and therapeutic purposes.

In conclusion, spontaneous recovery from acute HCV infection is associated with effective CD8^+^ T-cell responses. In contrast, progression to chronic persistent infection is associated with the impaired function of CD8^+^ T-cells and immune-evading viral mutations. Therefore, the induction of early and robust CD8^+^ T-cell responses will be a focus for development of successful HCV vaccines in the future.

## Author Contributions

Substantial contributions to the conception or design of the work; Pil Soo Sung, Vito Racanelli, Eui-Cheol Shin. Drafting the work or revising it critically for important intellectual content; Pil Soo Sung, Vito Racanelli, Eui-Cheol Shin. Final approval of the version to be published; Vito Racanelli, Eui-Cheol Shin.

## Conflict of Interest Statement

The authors declare that the research was conducted in the absence of any commercial or financial relationships that could be construed as a potential conflict of interest.
